# New Department

**Published:** 1883-06

**Authors:** 


### NEW DEPARTMENT.

In the July number we propose to organize a department in which may be brought to the notice of the profession whatever is new in dentistry; appliances, material, and remedies. It has too often been the case that the credit due to some patient investigator or experimenter has been filched from him and appropriated by another, perhaps even guarded by a patent, because of the lack of opportunity to properly claim priority of invention. We will undertake to receive any new appliance or invention, give it careful examination and trial, either personally or by some one of our associates, and report upon it through the pages of this journal, giving due credit to its originator. Thus the claim of originality may become a matter of imperishable record, and any dentist may secure the credit which may rightfully be his due.

We wish it distinctly understood that this is not to be a medium for indiscriminate puffery. We cannot undertake in all cases to even express a decided opinion upon the merits of every article submitted to our examination. But we will endeavor, when there is sufficient opportunity for fully testing any appliance, to give a fair presentation of any salient advantages or demerits which it may possess. The profession must trust to our honesty of purpose in giving our opinion, and we can assure it that we will not knowingly be biased in any way.

Articles will be returned at the expense of the owners, but full directions for such return must accompany them. They will be carefully used, but we cannot be responsible for any possible loss or damage.

